# Plasma Electrolytic Polished Patient-Specific Orbital Implants in Clinical Use—A Technical Note

**DOI:** 10.3390/jpm13010148

**Published:** 2023-01-11

**Authors:** Lara Schorn, Max Wilkat, Julian Lommen, Maria Borelli, Sajjad Muhammad, Majeed Rana

**Affiliations:** 1 Department of Oral-Maxillofacial and Facial Plastic Surgery, University Hospital Düsseldorf, Moorenstr. 5, 40225 Düsseldorf, Germany; 2 Department of Ophthalmology, University Hospital Düsseldorf, Moorenstr. 5, 40225 Düsseldorf, Germany; 3 Department of Neurosurgery, University Hospital Düsseldorf, Moorenstr. 5, 40225 Düsseldorf, Germany

**Keywords:** plasma electrolytic polished (PeP), orbital PSI, patient-specific implants, CAD/CAM, 3D printing

## Abstract

This technical note describes the technique of plasma electrolytic polishing on orbital patient-specific implants and demonstrates clinical handling and use by the insertion of a plasma electrolytic polished orbital implant into a patient.

## 1. Introduction

Severe trauma or ablative tumor surgery requires precise reconstruction in order to reestablish facial functions and aesthetics. Patient-specific implants (PSI) offer individual and exact options for reconstruction [1]. After 3D scanning (magnetic resonance imaging (MRI), computed tomography (CT) or cone beam computed tomography (CBCT)), PSIs are digitally planned and 3D printed using selective laser melting techniques (in case of titanium implants) [1,2]. Most often they are used for orbital wall reconstruction after trauma [2], but they are also frequently used for mandibular reconstruction [3] or even for orthognathic surgery [4]. In terms of orbital PSIs, the implants are planned mirroring the other, healthy, orbit. The gold standard material for PSIs remains titanium, but other materials such as PEEK (Polyetheretherketone) have recently been introduced [4,5]. During the usual manufacturing process, titanium patient-specific implants are machine polished. Machine polishing leaves the orbital implants sightly rough. The smoothness is limited due to the reductive process of machine polishing, eventually removing important structural detail of the already very thin orbital implant. A smooth surface minimalizes bacterial growth and reduces unwanted fibrozation [6]. The aim of orbital reconstruction is to regain normal ocular motility, to restore the orbital volume, and to relieve herniated tissues inducing minimal trauma [7]. For orbital reconstruction in particular, a very smooth implant surface is necessary for the surrounding tissues to glide freely along the implant. Scarring and unwanted fibrozation can severely impact the orbital motility, leading to double vision and resulting in secondary surgery [8]. Fractured orbital walls can be difficult to access and to denudate. The insertion and placement of large orbital PSIs might be very challenging without getting caught in the surrounding tissues. A smoother implant could ease insertion. Furthermore, in cases of secondary orbital reconstruction, implants with smooth surfaces might be easier to remove and reapply. A new technique, electropolishing the surface of titanium made PSIs, offers extremely smooth implant surfaces. Plasma electrolytic polishing (PeP) has successfully been used in the aerospace industry [9] and it has recently been suggested for medical use [10].

This technical note describes the technique of plasma electrolytic polishing (PeP) of orbital patient-specific implants and demonstrates its clinical applicability by using a PeP orbital PSI to reconstruct an orbital trauma patient.

## 2. Methods and Results

### 2.1. Plasma Electrolytic Polishing

Electropolishing is an electrochemical treatment resulting in leveling, cleaning, and surface finishing of a metallic surface. Electropolishing is consequently assigned to the electrically ablative manufacturing processes (DIN 8580). It is the electrochemical removal of a surface as a result of the electrical charge exchange between a metallic object and a liquid medium, the electrolyte. For PeP, anodic poled metals are placed into an electrolytic environmentally-friendly aqueous bath containing ammonium sulfate solution (95–98% H_2_O + 2–5% NH_3_). Here, a reverse galvanic process occurs. During submerging, a discharge process occurs at the anode. Punctual short circuits occur, leading to plasma development. The actual electrolysis processes take place in the resulting gas zone. Under the influence of the direct current, the electrolyte dissolves parts of the material surface. The surface is smoothed, leveled, and passivated. In addition, all organic and inorganic contaminants are removed with only minimal loss of material. Average material removal is 4–10 µm/minute, enabling leveling of micro-roughness (<0.01 µm). The geometric shape of the implant remains fully preserved [10,11,12,13].

### 2.2. Surgery

**Figure 1:**
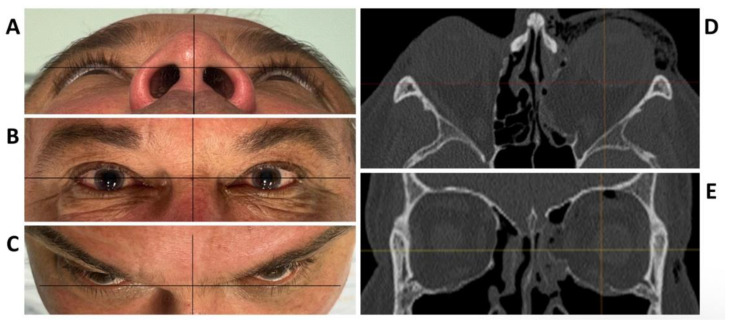
Clinical photographs of the patient showing an enophthalmos in the perspective from below (**A**), and above (**C**), while there is no hyper- or hypoglobe present as visible in the frontal view (**B**). Axial (**D**) and coronal (**E**) plane of the pre-operative CT scan displaying the traumatic defect of the medial wall and floor of the left orbit.

**Figure 2:**
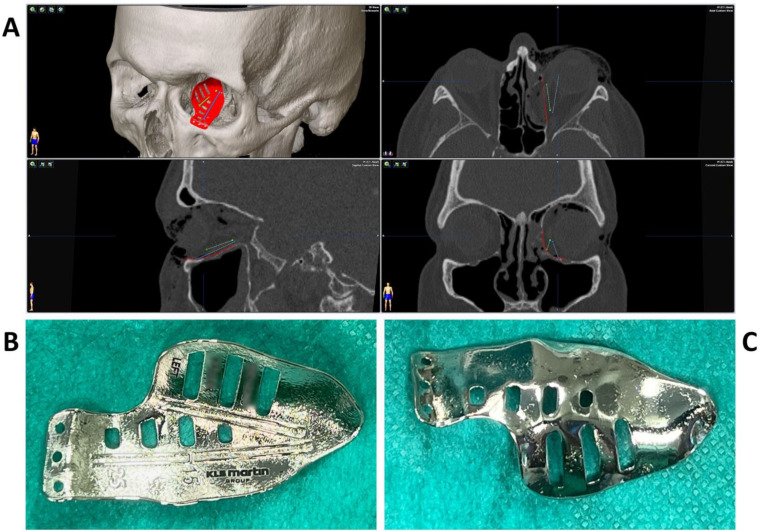
Multiplanar view of the pre-operative CT scan (**A**) with the finished computer-aided design of the patient-specific implant (red) for reconstruction of the damaged left orbit. Two trajectories (blue and green) have been defined which follow the grooves on the upper side of the implant (**B**) for position control via intraoperative navigation. The underside of the implant (**C**) shows a surface smoothened by plasma electrolytic polishing.

**Figure 3:**
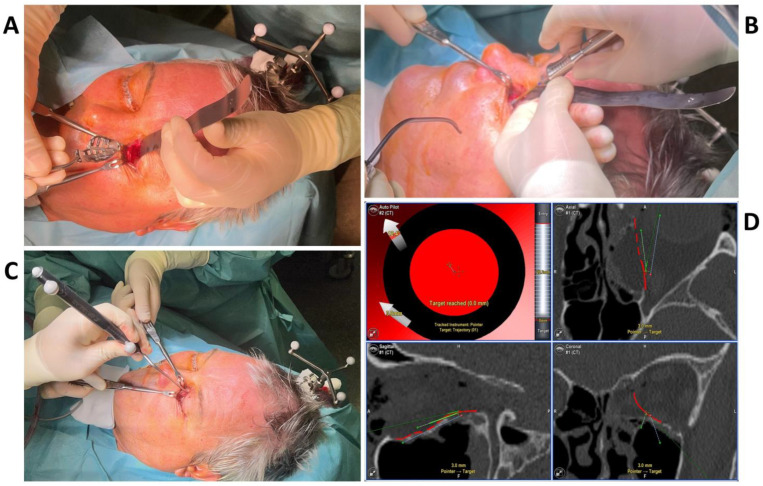
The intraoperative photographs show the preparation of the left orbit via a transconjunctival incision and insertion of the patient-specific implant (**A**), which is fixed with one 4 mm osteosynthesis screw at the infraorbital rim (**B**). Intra-operative real-time navigation using a navigation probe to follow the grooves on the implant (**C**) confirm the correct implant positioning by reaching the target of the set trajectories (**D**).

**Figure 4:**
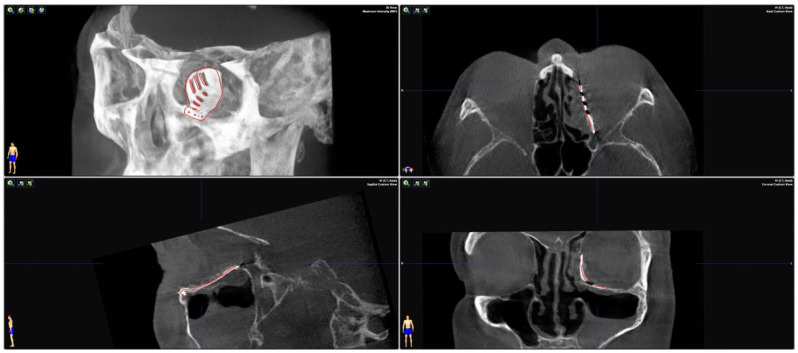
Merging the pre-operative planning CT data with the post-operative CBCT scan confirms correct positioning as the virtually planned patient-specific implant co-localizes with the inserted implant.

To test the applicability of the electropolished implant, we reconstructed the medial orbital wall and the orbital floor using a PeP orbital patient-specific implant in an orbital trauma case. The patient, male, 42 years old, presented himself to the emergency department of the University Hospital Düsseldorf with an enophthalmos and double vision after facial trauma (Figure 1A–C). In the CT imaging, fractures and large defects of the medial wall and the orbital floor of the left orbit were detected (Figure 1D,E). Accordingly, an orbital PSI was planned using computer-aided design techniques (iPlan, Brainlab, Feldkirchen, Germany (Figure 2A). The implant was produced by KLS Martin (Tuttlingen, Germany), showing a conventionally machine polished inner implant surface facing the orbit (Figure 2B), while the outer implant surface was smoothened by PeP (Figure 2C). Reconstruction of the orbit was performed using a transconjunctival approach (Figure 3A). After preparation of the medial orbital wall and the orbital floor, the implant was inserted and fixed with one 4 mm ostheosynthesis screw (Figure 3B). Insertion appeared to be easy, without soft tissue entanglement, and the supposed implant position could quickly be determined. The correct implant position was assured using intra-operative real-time navigation (Figure 3C,D). An additional post-operative CBCT scan confirmed correct positioning (Figure 4). Postoperatively, the patient showed no enophthalmos, double vision or ©mpairment of orbital mobility. Implant placement proved to be accurate and easy.

## 3. Discussion

Little is known about the impact of surface roughness of osteosynthesis plates for reconstruction on bone and soft tissue regeneration. However, the impact of different surfaces of dental implants in terms of their impact on osteointegration, periimplantitis, and gingival soft tissue is frequently discussed in the literature [6,14]. Osteosynthesis plates for craniofacial trauma are often removed after a healing period of 6 months [15]. Orbital PSIs and titanium meshes, however, are used for reconstructive purposes and are supposed to serve for a lifetime. The existence of a “titanium adhesion syndrome” which describes the adherence of orbital and periorbital structures on titanium meshes, resulting in diplopia and/or eyelid retraction, has been discussed in the literature [16,17]. However, more likely than the material itself causing these complications, the rough surface structure of the titanium meshes might be the reason. During tissue regeneration, cell behavior largely depends on material surface characteristics [18]. A rough implant surface increases cell accumulation, attracting fibrous tissue and bacteria [19,20]. Once inserted and healed, a misplaced implant is difficult to remove. Challenging conditions during implant placement, scarring, and muscular entrapment or soft tissue entanglement can lead to serious consequences, such as double vision or even blindness [21]. Eventually, secondary surgery becomes necessary [8]. An extremely smooth surface seems to be preferable if removal of an implant is indispensable. Compared to mechanical polishing, with PeP, the whole implant surface, or only parts of it, can be polished in only a few minutes [12,13]. The shape of the implant is not harmed, which is essential for accurate positioning. PeP of PSIs therefore offers not only advantages during clinical use, but helps patients’ recovery by allowing precise manufacturing and accelerating manufacturing processes.

In conclusion, PeP offers a new technique for the production of smoother patient-specific orbital implants without loss of important structural detail. These polished implants promise to be easier to apply and remove by inducing less orbital scarring. However, prospective studies have to prove the effect of a smoother implant surface on orbital tissues.

## Data Availability

The datasets used and/or analysed during the study are available from the corresponding author on reasonable request.

## References

[1] Gander T., Essig H., Metzler P., Lindhorst D., Dubois L., Rucker M., Schumann P. (2015). Patient specific implants (PSI) in reconstruction of orbital floor and wall fractures. J. Craniomaxillofac. Surg..

[2] Rana M., Moellmann H.L., Schorn L., Lommen J., Rana M., Wilkat M., Hufendiek K. (2022). Primary Orbital Reconstruction with Selective Laser Melting (SLM) of Patient-Specific Implants (PSIs): An Overview of 96 Surgically Treated Patients. J. Clin. Med..

[3] Lommen J., Schorn L., Sproll C., Haussmann J., Kubler N.R., Budach W., Rana M., Tamaskovics B. (2022). Reduction of CT Artifacts Using Polyetheretherketone (PEEK), Polyetherketoneketone (PEKK), Polyphenylsulfone (PPSU), and Polyethylene (PE) Reconstruction Plates in Oral Oncology. J. Oral. Maxillofac. Surg..

[4] Kerkfeld V., Schorn L., Depprich R., Lommen J., Wilkat M., Kubler N., Rana M., Meyer U. (2022). Simultaneous PSI-Based Orthognathic and PEEK Bone Augmentation Surgery Leads to Improved Symmetric Facial Appearance in Craniofacial Malformations. J. Pers. Med..

[5] Lommen J., Schorn L., Sproll C., Kubler N.R., Nicolini L.F., Merfort R., Dilimulati A., Hildebrand F., Rana M., Greven J. (2022). Mechanical Fatigue Performance of Patient-Specific Polymer Plates in Oncologic Mandible Reconstruction. J. Clin. Med..

[6] Rothamel D., Heinz M., Ferrari D., Eissing A., Holtmann H., Schorn L., Fienitz T. (2022). Impact of machined versus structured implant shoulder designs on crestal bone level changes: A randomized, controlled, multicenter study. Int. J. Implant. Dent..

[7] Ellis E., Messo E. (2004). Use of nonresorbable alloplastic implants for internal orbital reconstruction. J. Oral. Maxil. Surg..

[8] Singh D.D., Schorn L., Strong E.B., Grant M., Schramm A., Hufendiek K., Gellrich N.C., Rana M. (2021). Computer-Assisted Secondary Orbital Reconstruction. Craniomaxillofac. Trauma. Reconstr..

[9] Navickaite K., Nestler K., Böttger-Hiller F., Matias C., Diskin A., Golan O., Garkun A., Strokin E., Biletskiy R., Safranchik D. (2022). Efficient polishing of additive manufactured titanium alloys. 6th CIRP Conference on Surface Integrity.

[10] Kröning O., Schulze H.-P., Zeidler H., Kranhold C. (2019). Das Plasma-elektrolytische Polieren von Werkstücken für den medizin- technischen Einsatz. Magdebg. Ing..

[11] Plasotec G. Verfahren. https://plasotec.de/verfahren.html.

[12] Danilov I., Hackert-Oschatzchen M., Zinecker M., Meichsner G., Edelmann J., Schubert A. (2019). Process Understanding of Plasma Electrolytic Polishing through Multiphysics Simulation and Inline Metrology. Micromachines.

[13] Cornelsen M., Deutsch C., Seitz H. (2017). Electrolytic Plasma Polishing of Pipe Inner Surfaces. Metals.

[14] Novaes A.B., de Souza S.L., de Barros R.R., Pereira K.K., Iezzi G., Piattelli A. (2010). Influence of implant surfaces on osseointegration. Braz Dent. J..

[15] Danielis J., Albakry I., Braimah R., Samara M. (2021). Is the Routine Removal of Titanium Plates and Screws Following Miniplate Osteosynthesis of Maxillofacial Bone Fractures Justified? A Fifteen-Year Experience in a Maxillofacial Centre, Saudi Arabia. Craniomaxillofacial. Res. Innov..

[16] Lee G.H.P., Ho S.Y.M. (2017). Orbital Adherence Syndrome following the Use of Titanium Precontoured Orbital Mesh for the Reconstruction of Posttraumatic Orbital Floor Defects. Craniomaxillofacial. Trauma Reconstr..

[17] Sleem H., Wahdan W. (2018). Orbital adherence syndrome: Clinical characterization and risk factor tracing (retrospective clinical research). Egypt. J. Oral. Maxillofac. Surg..

[18] Trajkovski B., Jaunich M., Muller W.D., Beuer F., Zafiropoulos G.G., Houshmand A. (2018). Hydrophilicity, Viscoelastic, and Physicochemical Properties Variations in Dental Bone Grafting Substitutes. Materials.

[19] Dohan Ehrenfest D.M., Coelho P.G., Kang B.S., Sul Y.T., Albrektsson T. (2010). Classification of osseointegrated implant surfaces: Materials, chemistry and topography. Trends. Biotechnol..

[20] Burgers R., Gerlach T., Hahnel S., Schwarz F., Handel G., Gosau M. (2010). In vivo and in vitro biofilm formation on two different titanium implant surfaces. Clin. Oral. Implant. Res..

[21] Schlittler F., Vig N., Burkhard J.P., Lieger O., Michel C., Holmes S. (2020). What are the limitations of the non-patient-specific implant in titanium reconstruction of the orbit?. Br. J. Oral. Maxillofac. Surg..

